# When migrants do not speak the host country’s language but need mental health care: A protocol for developing a communication intervention

**DOI:** 10.1371/journal.pone.0338040

**Published:** 2025-12-05

**Authors:** Houda Al Kalaf, Christopher Jenks, Mike Mösko, Ted Sanders, Barbara Schouten

**Affiliations:** 1 Department of Languages, Literature and Communication, Utrecht University, Utrecht, The Netherlands; 2 Department of Medical Psychology, University Medical Center Hamburg Eppendorf, Hamburg, Germany; 3 University of Applied Science Magdeburg-Stendal, Stendal, Germany; 4 Faculty of Social and Behavioural Sciences, ASCoR, Center for Urban Mental Health, University of Amsterdam, Amsterdam, The Netherlands; PLOS: Public Library of Science, UNITED KINGDOM OF GREAT BRITAIN AND NORTHERN IRELAND

## Abstract

Migrant clients frequently encounter communication barriers in mental healthcare, primarily driven by language differences that complicate the expression and understanding of psychological distress. Language barriers also affect mental healthcare providers (MHCPs), who often report low confidence and limited skills in navigating cross-linguistic encounters. Inadequate access to trained interpreters, uncertainty about their roles, and a lack of structured training leave MHCPs underprepared to ensure accurate communication and offer person-centred care. Strong collaboration between MHCPs, clients, and interpreters can significantly reduce communication barriers, fostering shared understanding and trust. However, existing initiatives to address language-related challenges in mental healthcare for migrants remain fragmented, lack cultural sensitivity, and are rarely designed or evaluated systematically.

This study addresses this need by designing, developing, implementing, and evaluating a structured communication intervention that aims to strengthen MHCPs’ ability to provide care for migrant clients who face language barriers. The intervention mapping (IM) framework is used to conduct an extensive needs assessment, including interviews with MHCPs, clients, and interpreters across multiple countries, to identify communication challenges and inform intervention design. The training program was co-developed with stakeholders and pilot-tested to assess clarity, engagement, and practical relevance. The resulting evidence-informed program seeks to improve therapeutic communication and ultimately support better mental health outcomes for linguistically diverse migrant populations.

**Protocol Registration:**

This study protocol has been preregistered with the Open Science Framework (OSF) to enhance transparency, reproducibility, and methodological rigour. The registration includes a detailed description of the study objectives, design, intervention components, target population, outcome measures, and planned analyses. Any future amendments to the protocol will be documented and timestamped on the OSF platform to maintain a clear audit trail of changes. The registration is publicly accessible via OSF https://osf.io/g2jk7.

## Introduction

Effective communication is fundamental to high-quality mental healthcare and essential for person-centred care approaches. A common barrier to effective communication is when clients and mental healthcare providers (MHCPs) do not share a common language [1–3]. Language barriers adversely affect care [4] because cultural nuances embedded in language may lead to misunderstandings, misinterpretation of symptoms, and errors in clinical decision-making, ultimately compromising patient safety, engagement, and treatment outcomes [5]. For instance, physicians tend to adopt an authoritarian, biomedical-focused approach that ignores clients’ personal circumstances in decision-making processes if they do not understand or appreciate how a patient from a different language background communicates health issues [5]. Variations in health beliefs and communication styles adversely affect critical health outcomes, including patient safety, medical adherence, continuity of care, and physicians’ ability to establish credibility and make accurate diagnoses [6].

In the case of migrants, communication barriers are compounded by issues of vulnerability; for example, refugees are exposed to a variety of stressors that significantly affect their mental health and overall well-being before, during, and after migration [7]. Such stressors include trauma, displacement, loss, socio-economic instability, and difficulties adjusting to new environments. Additionally, migrants encounter barriers when seeking mental health care because of language difficulties, cultural differences, limited knowledge of services, stigma, and complex legal or administrative requirements [8]. Even when care is sought, health professionals struggle to understand the unique cultural and communicative challenges of providing mental healthcare to migrants, potentially hindering treatment efficacy [9]. Furthermore, specialised mental health services for migrants suffer from poor quality, lacking language support, culturally adapted treatments, and professionals with intercultural competencies [10]. These shortcomings in services, along with inadequate training in effective communication, often lead to alienation, disengagement, and worsened mental health outcomes [11,12]. While all these barriers to care are significant, such as limited service availability, mental health stigma, lack of culturally adapted care, and systemic or legal obstacles, language barriers are particularly important because they directly impede the core component of the therapeutic process: communication. Addressing communication difficulties leads to better understanding, stronger rapport, and more effective care, which can help mitigate other barriers to mental healthcare that migrants face. Indeed, improving MHCPs’ communication skills in mitigating language barriers is associated with better patient satisfaction and treatment adherence [13].

Collaboration with interpreters is essential for overcoming language barriers in mental healthcare settings. Effective partnerships between healthcare professionals and interpreters ensure accurate diagnoses, effective treatment planning, and the establishment of rapport with clients [14,15]. In interpreter-mediated interactions, the interpreter serves as a crucial communication bridge, though it is important to establish clear roles and expectations between the provider, interpreter, and patient to ensure successful interaction [16]. MHCPs must also employ positive, warm, and empathetic communication, which, when combined with realistic expectations, can alleviate patient anxiety, improve mood, and enhance overall satisfaction [17]; this approach is particularly important in overcoming language barriers, as effective communication fosters a sense of being understood and supported, thereby mitigating the additional challenges introduced by language differences [11,18,19].

Across Europe, ongoing efforts are focused on developing training programs and resources to improve mental healthcare for migrants facing language and cultural barriers [2,3,20–25]. However, most programs do not adequately address the communication challenges faced by migrant clients during mental healthcare encounters. Furthermore, many programs do not use rigorous evaluation tools to assess their effectiveness (Al-Kalaf et al., manuscript in preparation). This study addresses this need by presenting a protocol for an evidence-based communication intervention that specifically addresses language barriers in mental health settings. Our goal is to help MHCPs communicate effectively with migrant clients with low language proficiency in the home/ dominant language(s) of their host country. We will conduct a process evaluation alongside assessing the intervention’s impact, examining both its implementation and effectiveness in enhancing the MHCPs’ communication skills in linguistically challenging encounters.

### Study goal, objectives, and research questions

The goal of this study is to develop, implement, and evaluate a communication intervention aimed at improving the self-reported quality of mental healthcare communication between MHCPs and migrant clients with limited proficiency in the home/ dominant language(s) of their host country. Specifically, the intervention seeks to enhance MHCPs’ capacity to navigate linguistically challenging clinical encounters, including those that involve professional and non-professional interpreters [15,16,26].

Communication interventions in mental healthcare focus on addressing persistent barriers that hinder effective interaction between MHCPs and clients. These barriers include the misinterpretation of clinical information, clients’ challenges in expressing psychological or emotional distress, limited client participation in decision-making, and ambiguity surrounding the roles and responsibilities of interpreters [2,19,27]. When left unaddressed, such barriers compromise care quality, reduce client engagement, and exacerbate health disparities among migrant populations [4,8,11]. By equipping MHCPs with practical tools and strategies to manage communication in these contexts, the intervention seeks to facilitate accurate, culturally and linguistically responsive care [14,17].

The design and implementation of the intervention will follow a participatory planning approach [28]. This process includes active collaboration with local stakeholders to ensure that the intervention is contextually relevant and responsive to the specific needs of diverse migrant populations. The intervention is grounded in theoretical models of behavioural change and draws on empirical evidence from previous research on cross-cultural and interpreter-mediated communication in mental healthcare settings [20,21,23].

This protocol pertains to the Dutch intervention, situated within a broader international project. A communication intervention will be implemented in Germany, Romania, and South Africa; however, its design and delivery will be adapted to the specific linguistic, cultural, and systemic contexts of each country. Accordingly, this document outlines the planned intervention for the Netherlands.

### Research objectives

1. To identify communication barriers and interactional processes that occur during linguistically complex mental healthcare encounters with migrant clients, focusing on how MHCPs, clients, and interpreters collaborate and negotiate understanding in interpreter-mediated consultations.
2. To co-design a structured training intervention with stakeholders that addresses identified gaps and strengthens collaborative communication practices.
3. To pilot-test the intervention to assess its comprehensibility, engagement, credibility, and relevance for improving MHCPs’ communication skills in real-world mental healthcare settings.

### Research questions

To meet the above objectives, we will respond to the following research questions:

1. What language-related barriers and interactional challenges do MHCPs, interpreters, and migrant clients face during multilingual mental healthcare consultations, and how do they collaborate and negotiate understanding in these encounters?
2. Which components and delivery methods should be integrated into a feasible and contextually relevant training intervention to improve communication practices?
3. To what extent is the developed communication training intervention perceived by MHCPs as comprehensible, engaging, credible, and relevant for application in their multilingual mental healthcare encounters?

## Methodology

The intervention uses intervention mapping (IM) [28] to systematically describe the content and planning of complex interventions [29]. IM is an iterative process, starting with problem identification and ending with problem-solving by means of behavioural and environmental change programmes.

IM encompasses six steps [28]. The planning process starts with a needs assessment in step 1, resulting in a ‘logic model of the problem’, which describes the health problem, its impact on quality of life, behavioural and environmental causes, and determinants of these causes. In step 2, planners identify target groups and prioritise important and changeable objectives. The product is composed of behavioural outcomes (desired behaviours of all actors), performance objectives (breakdown of behavioural outcomes), and change objectives (determinants of the performance objectives). In step 3, planners select behavioural and environmental change methods and practical applications. Methods are theory-based processes influencing change in determinants. Practical applications are operational translations of methods that fit the intervention context and target population and are developed along the evidence-based working mechanisms (parameters of use) of the method. In step 4, the integration of applications results in a coherent programme with pilot-tested intervention materials. For programme adoption, implementation, and sustainability, planners design an implementation intervention as step 5. Finally, in step 6, planners design an evaluation plan to investigate the extent to which the intervention has been implemented as intended and has reached its objectives.

### Study design

#### Step 1: The results of the needs assessment.

A qualitative pilot study [30] identified multi-level barriers that low language proficient migrants face in accessing mental health services in the Netherlands, Germany, South Africa, Romania, and China. Interviews were conducted with community and policy representatives, mental healthcare workers, and migrant clients of different nationalities. Among the challenges identified, language barriers emerged as a predominant issue affecting mental healthcare for migrants in these countries. To address this, interpreters, digital tools, and multilingual staff were employed. Notably, the study highlighted significant inadequacies and a lack of professional interpretation services in the healthcare systems. Based on participants’ experiences, the study underscores the need for interventions that address linguistic barriers in mental healthcare.

In addition, a systematic literature review(Al-Kalaf et al., manuscript in preparation) synthesised empirical findings regarding communication challenges, strategies, and solutions in mental health care for migrants with language barriers. The results show that language barriers are a significant obstacle in providing mental health services to migrants. Language disparities between clients and providers can adversely influence clinical evaluations and treatments, potentially leading to poorer health outcomes for clients. Often, interpreting services are unavailable, and when they are, interpreters may lack the specialised training required for mental health counselling, especially for sensitive populations like refugees and migrants. Moreover, MHCPs frequently do not possess the necessary training to collaborate effectively with various interpreter services. While the literature does offer some practical advice on training to enhance the capabilities of mental health staff working with refugees and migrants, these initiatives are not often systematically implemented and lack a research-based approach to pinpoint factors that could enhance the execution, continuity, and success rate of such interventions.

Furthermore, an interview study was conducted by the intervention countries. Each country partner conducted interviews with MHCPs, mental health clients, formal and informal interpreters, and patient supporters. In the Netherlands, interviews were held with clients from Moroccan and Syrian backgrounds. The results of the interviews provided critical insights that informed the intervention’s content and design (Al-Kalaf et al., manuscript in preparation).

Notably, the interview study identified communication barriers, such as challenges in conveying emotions, medical terminology, and cultural nuances, which are particularly relevant when working with migrant clients. These barriers were found to compromise the accuracy of assessments, hinder therapeutic rapport, and reduce overall treatment effectiveness. Additionally, MHCPs reported low levels of confidence and competence in managing linguistically diverse and culturally complex cases, highlighting the need for targeted training to improve the self-efficacy of mental health care providers. The interviews also revealed the preferred communication strategies of migrant clients, such as the use of online resources, visual aids, and simplified language. This information informed the design of our intervention aimed at enhancing communication accessibility. Another key finding was that both clients and providers often had an unclear understanding of the role of interpreters, whether professional or informal. The study highlighted the need to clarify the role of interpreters in ensuring accurate and culturally sensitive communication; the design of the current intervention attended to address these misconceptions and expectations.

Based on the results of the needs assessment, we classified interpreting services in mental healthcare into three main categories: professional interpreters, ad hoc interpreters, and family members or friends acting as informal interpreters. *Professional interpreters*, who have completed specialised training [31] and are compensated for their work, are often employed by either a healthcare institution or a dedicated language service. Alternatively, interpretation might be carried out by *ad hoc interpreter*s. These are bilingual individuals who are proficient in the patient’s language [32], possibly nonclinical or clinical staff within the hospital, who lack formal training in interpretation but are called upon to facilitate communication [31]. While this approach is practical, the quality of interpretation will deviate from that provided by seasoned professionals with extensive experience. In some instances, interpreting is even conducted by *informal interpreters*, such as family members or friends who often possess a marginally better understanding of the MHCPs’ language than the patient [33], but have no formal training in medical interpretation. However, an advantage of informal interpreters is that they are often aware of the patient’s lived experiences and can give the provider background information that can be beneficial for treatment. This classification informed the intervention’s scope and guided the design of training components addressing the unique challenges and dynamics of working with each type of interpreter.

**Potential Programme Beneficiaries.** For the Dutch intervention, the programme will target MHCPs as its primary beneficiaries. This group is defined broadly to reflect the variety of professionals involved in mental health care in the Netherlands and may include clinical psychologists, psychiatric nurse practitioners, social workers, and other relevant providers. A purposive sample of at least 20 participants will be recruited through professional networks, institutional partners, and community organisations, and subsequently divided into two training groups of approximately 10 participants each.

#### Step 2: Matrices of change objectives.

**Performance Objectives.** The main goal of the intervention is to improve MHCPs’ self-reported communication competencies in clinical encounters when there is no common language. Performance objectives (see Table 1) related to this aim are based on person-centred care strategies that consider individual clients’ needs, values, and preferences to reach a common ground between migrant clients and their mental healthcare providers. After the intervention, MHCPs are expected to report improved communication skills for overcoming language barriers and enhanced ability to work effectively with various types of interpreters compared to their abilities before the intervention. Providers will also gain skills to use alternative strategies when interpreters are unavailable or to complement their efforts. These include using digital tools, printed materials, non-verbal communication, drawings, multilingual information sheets, and clear, slow speech to help bridge language barriers.

**Table 1:** The performance objectives.

Beneficiaries	Performance objectives
1. Mental health providers	1.1 After the intervention, MHCPs’ have improved self-reported communication competencies to work effectively with professional interpreters in linguistically challenging clinical encounters.
	1.2 After the intervention, MHCPs’ have improved self-reported communication competencies to work effectively with ad-hoc interpreters in linguistically challenging clinical encounters.
	1.3 After the intervention, MHCPs’ have improved self-reported communication competencies to work effectively with informal interpreters such as family and friends in linguistically challenging clinical encounters. 1.4 After the intervention, MHCPs’ have improved self-reported competencies to use alternative strategies, e.g., translation applications and online tools to mitigate language barriers.

**Determinants and Change Objectives.** The needs assessment indicates that MHCPs frequently report low self-efficacy regarding their communication abilities when engaging with migrant clients who speak a language different from their own [2,34]. Additionally, MHCPs expect to receive support from certain members of their clinics and colleagues at work when needed [35]. Addressing social norms is crucial because they shape behaviours, influence workplace culture, and can either enable or hinder help-seeking and collaboration among MHCPs, ultimately impacting the effectiveness of interventions [36]. The needs assessment further emphasises that MHCPs often lack adequate knowledge regarding the use of different types of interpreters and communication strategies, which impedes effective communication with migrant clients [26,37].

To guide the intervention’s development, the theory of planned behaviour (TPB) [38] provides the guiding framework for understanding and influencing MHCPs’ communication in linguistically challenging encounters. Drawing on the findings from the needs assessment, three key determinants within the TPB were selected to guide the change objectives: knowledge, perceived social norms, and self-efficacy. *Knowledge* refers to MHCPs’ understanding of effective communication strategies in linguistically challenging encounters, including cultural responsiveness and role clarity when working with professional, ad hoc, and informal interpreters. Although not a core construct in TPB, knowledge is considered foundational, as it supports the formation of behavioural intention [39]. *Perceived social norms* reflect MHCPs’ beliefs about others’ expectations, such as colleagues, supervisors, and their professional community, regarding how to effectively communicate with linguistically diverse clients. Enhancing these norms helps to foster a sense of professional responsibility and social support for engaging in culturally and linguistically responsive communication [38,40]. *Self-efficacy* pertains to MHCPs’ confidence in their ability to manage communication challenges in diverse linguistic settings, including interactions involving interpreters. Strengthening self-efficacy is critical to ensuring that MHCPs feel competent and prepared to apply the skills and strategies learned in training [41,42].

These three determinants serve as the foundation for the intervention’s change objectives, which specify what needs to change in each area to promote improved communication between MHCPs and clients with limited proficiency in the dominant language(s) of care.

Table 2 presents the matrix of change objectives to reflect the necessary changes in knowledge, perceived social norms, and self-efficacy in the target audience to achieve the four performance objectives outlined above.

**Table 2:** The intervention’s change objectives.

Beneficiaries	Performance objectives (PO)	Change objectives		
		Knowledge	Perceived social norms	Self-efficacy
**Mental healthcare providers**	Improved self-reported communication competencies, resulting in effective collaboration with professional interpreters in linguistically challenging clinical encounters.	Providers know the strategies that are necessary to work with professional interpreters in mental health settings	Providers express that they can get support from their peers and supervisors to work effectively with professional interpreters to mitigate language barriers	Providers perceive themselves to have the skills to be able to work effectively with professional interpreters during encounters where there are language barriers present
	Improved self-reported communication competencies, resulting in effective collaboration with ad-hoc interpreters in linguistically challenging clinical encounters.	Providers are aware that working with ad-hoc interpreters is different from other types of interpreters. They know the strategies that are necessary to work with ad-hoc interpreters in mental health settings	Providers express that they can get support from their peers and supervisors to work effectively with ad-hoc interpreters to mitigate language barriers	Providers perceive themselves to have the skills to be able to work effectively with ad-hoc interpreters during encounters where there a are language barriers present
	Improved self-reported communication competencies, resulting in effective collaboration with informal interpreters such as family and friends in linguistically challenging clinical encounters.	Providers are aware that working with informal interpreters is different from other types of interpreters. They know the strategies that are necessary to work with family and friends’ interpreters in mental health settings	Providers express that they can get support from their peers and supervisors to work effectively with family/friends interpreters to mitigate language barriers	Providers perceive themselves to have the skills to be able to work effectively in collaborating with informal interpreters during encounters where there are language barriers present
	Improved self-reported communication competencies, resulting in effective use of communication alternative strategies in linguistically challenging clinical encounters.	Providers are aware of the different alternative strategies that they can use to help substitute/support interpreters in bridging linguistic barriers	Providers express that they can get support from their peers and supervisors to work effectively with alternative strategies to mitigate language barriers	Providers perceive themselves to have the skills to use alternative strategies effectively during encounters where there are language barriers present

#### Seps 3 and 4: Methods, applications, and programme design.

Steps 3 and 4 were conducted simultaneously, during which we selected the intervention methods and applications and designed the programme, ensuring both were directly linked to the change objectives identified in Step 2. Table 3 provides theory-based methods and definitions for each change objective. Practical applications and their parameters are also included, as they are crucial factors that affect the way we apply theory to practice and ensure the effectiveness of the method.

**Table 3:** Methods and applications for change objectives per behavioural determinant.

Determinant	Theoretical method	Definition of the action	Parameters for use	Practical applications
Knowledge	Reflective-Impulsive Model [43]	Stimulating the learner to add meaning to the information that is processed.	Individuals with high motivation and high cognitive ability; messages that are personally relevant, surprising, repeated, self-pacing, not distracting, easily understandable, and include direct instructions; messages that are not too discrepant and cause anticipation of interaction	*Before the training:* E-learning modules will provide essential knowledge about confidentiality and role expectations. *During the training,* the trainer presents case studies and relevant information, encouraging participants to engage in critical thinking. By posing challenging questions and discussing realistic scenarios, the trainer prompts participants to deeply analyse the information, fostering a thorough understanding of effective communication strategies for navigating linguistic barriers in mental health care. *During the booster session:* MHCPs will share the updates and improvements they have made to their acquired knowledge.
Perceived social influence	Social norms interventions [44]	Establish a clear understanding of shared values and professional expectations by utilising injunctive norms to mobilise peer influence among mental health workers to work effectively with different types of interpreters. By highlighting what is socially approved within the group, participants are more likely to align their behaviour with these norms	Ensure influential peers are respected and trusted within the group. Regularly reinforce positive behaviours and challenge negative ones through rewards, recognition, and awareness. Availability of social network and potential support givers. Will often include information about others’ approval, facilitation and persuasive communication.	*During the training:* Participants are encouraged to actively discuss their needs with supervisors and peers, envisioning and practicing the support they require to enhance their communication skills. Additionally, the trainer presents testimonial messages from influential role models, inspiring participants to apply the newly acquired knowledge in their professional interactions. Social support: MHCPs will be encouraged to establish support networks to share experiences and motivate each other. This will play an important role in fostering positive social norms. *After the training:* Participants can contact the trainer or research team for support. *The booster session* will provide a platform for participants to share experiences and success stories with one another.
Self-efficacy	Social Cognitive Theory [45,46].	Prompt planning as to what the beneficiaries will do, including a definition of goal-directed behaviours that result in the target behaviour.	Group cohesion supports high levels of identification, which will lead to more effects on self-efficacy compared to lower levels of identification.	During the training: Modelling: Role-pays are developed to provide MHCPs with a safe environment to practice their skills and boost their self-efficacy. MHCPs will observe colleagues successfully navigating communication scenarios with different interpreters. Skills building: Educational materials will equip MHCPs with the knowledge and skills to effectively communicate with migrant clients, enhancing self-efficacy. Positive reinforcement: MHCPs will receive positive feedback from peers, further boosting their self-efficacy. Additionally, during the *booster session*, MHCPs are expected to further strengthen their self-efficacy by sharing experiences. Learning about others’ success in similar situations can enhance confidence in one’s own abilities.

**The Intervention:** The intervention is a structured communication training programme designed to enhance MHCPs’ ability to navigate linguistically challenging clinical encounters with migrant clients. The training focuses on building knowledge, shaping perceived social norms, and strengthening self-efficacy for working effectively with professional, ad-hoc, and informal interpreters, as well as using alternative communication strategies.

The communication intervention consists of:

- Two in-person interactive sessions as outlined in S1 Appendix (totalling six hours), delivered to two separate groups of participants, with sessions spaced approximately one week apart. Each session combines educational slides, real-world case studies, and structured role-plays, co-facilitated by two trainers.
- A 30-minute e-learning module, completed before the sessions, introduces the interpreter’s role, principles of confidentiality, and bias awareness. It also offers an overview of factors that may shape communication across different cultural and linguistic backgrounds. The module includes short quizzes and provides digital access to materials for later reference.
- An online booster session is held one month after training to allow reflection on the real-world application of learned skills and to promote sustained behavioural change [47].

**Theoretical Underpinnings:** As shown in Table 3, to achieve the first change objective related to “Knowledge”, we utilised the reflective-impulsive model [43]. This model explains how individuals process information through two distinct systems: the reflective system and the impulsive system. The reflective system involves deliberate, conscious processing of information, requiring cognitive effort and self-regulation, while the impulsive system is more automatic, driven by immediate emotional responses and environmental cues. According to the model, both systems influence behaviour, but the reflective system typically leads to more enduring and rational attitude changes [43,48].

To engage the reflective system, the trainer provides detailed, evidence-based information, encouraging participants to critically assess and reflect on the material [49]. Scenarios of case studies stimulate deep analysis and encourage participants to connect the information to real-world contexts, fostering cognitive processing and reflection [50]. The training content is further connected to participants’ professional goals through relevant case discussions and reflection activities to enhance motivation and facilitate the reflective processing of new information [51].

To address the second change objective related to “Perceived Social Norms”, the intervention employs social norms strategies informed by Prentice’s [44] work. She identifies two distinct types of norms: descriptive norms, which reflect typical behaviours within a group, and injunctive norms, which emphasise behaviours that are socially approved or disapproved. While descriptive norms illustrate what people commonly do, injunctive norms are shown to have greater efficacy in motivating behaviour because individuals are strongly influenced by the perceived approval or disapproval of their peers [44]. Within professional environments, these norms can act as powerful motivators for adopting desirable practices, including effective communication with interpreters to enhance patient care outcomes [52].

The intervention will utilise injunctive social norms. Specifically, the training highlights the professional and institutional endorsement practices such as engaging professional interpreters, speaking in clear and simple language, using visual aids or multilingual materials, and checking patient understanding, signalling that such behaviours are both expected and valued within the mental health care community. By making these normative expectations explicit, the intervention seeks to create a sense of social accountability and collective commitment. This use of normative influence is grounded in research showing that behaviours perceived as socially approved are more likely to be internalised and sustained in professional settings [53,54].

To apply this social norm approach, the intervention incorporates case studies and slides as tools to illustrate scenarios where MHCPs interact with interpreters to address the needs of migrant clients. These materials highlight specific strategies for successful communication and emphasise how such practices align with the professional norms of the healthcare community. By demonstrating that effective collaboration with interpreters is regarded as a mark of professional competence, the intervention reinforces the notion that these behaviours are both valued and expected within the field.

To achieve the third and final change objective related to “Self-Efficacy”, the intervention will apply social cognitive theory (SCT) [45,46]. This theory provides a framework for understanding behaviour change by emphasising the interplay between personal, behavioural, and environmental factors. A key construct within SCT is self-efficacy, or an individual’s belief in their ability to successfully execute the behaviours required to achieve desired outcomes [55]. Self-efficacy impacts the effort individuals exert, their persistence in overcoming challenges, and their resilience in the face of adversity. SCT identifies four principal sources of self-efficacy: mastery experiences, vicarious experiences (modelling), verbal persuasion, and physiological and emotional states [45].

The intervention employs SCT principles by integrating role-plays as the primary method for developing MHCPs’ self-efficacy. Role-playing, a pedagogical technique closely aligned with SCT, offers an effective means of fostering self-efficacy. Mastery experiences are cultivated as participants engage in structured role-play scenarios, successfully applying newly learned communication strategies in realistic settings. Modelling, another core SCT element, occurs as MHCPs observe trainers or peers effectively navigating challenging scenarios involving linguistic barriers. These observations provide vicarious experiences, demonstrating that success is achievable and offering a roadmap for replicating effective behaviours. Verbal persuasion, delivered as constructive feedback during and after role-plays, reinforces confidence by highlighting strengths and providing actionable improvements. Finally, role-plays create a controlled and supportive environment that reduces anxiety and fosters positive emotional states, further enhancing self-efficacy [45,56]

**Pilot Testing of the Training Materials:** Prior to full-scale implementation, a pilot study was conducted to assess the training materials designed for MHCPs. The aim was to ensure that all programme components were comprehensible, engaging, credible and relevant in multilingual mental healthcare encounters [57,58].

A purposive sample of 10 MHCPs currently working with migrant clients in linguistically challenging settings was recruited through professional networks. Recruitment for the pilot participants began on 1 April 2025 and concluded on 25 April 2025. Participants were provided with an information letter outlining the purpose and process of the pilot; no informed consent was required. Participants reviewed educational slides, case studies, and role-play scenarios independently. One week later, structured one-on-one feedback sessions were held to collect both quantitative and qualitative data on the materials.

Evaluation criteria included: (a) comprehensibility, referring to the clarity of instructions and concepts [59]; (b) engagement, assessing whether materials stimulated interest and active learning [51]; (c) credibility, examining whether examples and scenarios reflected authentic and culturally appropriate clinical situations [60]; and (d) relevance, determining the extent to which the training addressed MHCPs’ real-world communication challenges [61].

Feedback was analysed descriptively and thematically [62] to identify potential refinements. Insights from this pilot informed revisions to the training content, ensuring that the final intervention was clear, context-sensitive, and well aligned with MHCPs’ professional needs.

#### Step 5: Planning adoption, implementation and sustainability.

The aim of step 5 is to anticipate the programme adoption, implementation, and sustainability for users: community-based volunteers, providers, training staff, decision-makers, and leaders on all levels.

**Adoption and Implementation**. In the Netherlands, adoption of the intervention begins with the sensitisation of mental health care providers, non-governmental organisations, regional stakeholder groups, and decision-makers. Relevant stakeholders are identified based on the organisation of the Dutch mental health system, existing professional networks, and the specific needs and priorities of migrant populations to ensure contextual alignment and to maximise uptake [63,64]. This tailored approach supports the introduction and implementation of the intervention in a manner consistent with national infrastructures and priorities. The adoption and implementation groups consist of professionals who have experience working with migrant clients; these experiences are expected to inform their attitudes and promote supportive practices towards this population [65]. Subsequently, these participants will be involved as implementers who are willing to take a leading role in the intervention activities.

***Adoption Stage.*** The adoption stage focuses on engaging clinic decision-makers with the intervention. To begin the adoption process, the clinic decision-makers will receive emails that provide information about the intervention program. This initial communication helps gauge interest and begins educating staff about the benefits and logistics of the program [66].

If needed, an online webinar can be organised for clinic staff, providing key details about the intervention, including its structure, implementation steps, expected outcomes, and how it aligns with the clinic’s goals [67].

Additionally, a role modelling approach can be implemented by inviting someone who has already undergone the training during the pilot phase to attend an adoption meeting. This individual will share their personal experience and provide insights into the program’s impact. This real-world example will help clarify any doubts and encourage adoption [41]. Support will also be provided in terms of connecting clinic decision-makers with local agents, such as inviting stakeholders to join a group that can assist in facilitating the adoption process [68].

***Implementation Stage.*** The implementation stage focuses on encouraging active participation from all relevant stakeholders and providing the tools necessary for effective program delivery.

Once participation is confirmed, a site visit can be scheduled. A detailed email template will be sent to clinic staff, outlining the visit’s purpose, the participants needed, and the benefits of the visit for improving implementation efforts. During these site visits, on-the-ground planning meetings will be held to address any immediate concerns and discuss how the program can be smoothly integrated into existing clinic operations [66].

***Implementation (Program Champion Navigator).*** The program champion is a staff member who supports the implementation of the intervention by promoting engagement, coordinating logistics, and offering on-site support during the training [69]. The program champion navigator will take a leading role in driving the program’s implementation. They will be responsible for ensuring that the training and support mechanisms are in place and effective [66].

Ongoing implementation support will be provided on-site: a member of the research team will be present during training to offer real-time guidance, answer questions, and provide assistance as needed. This hands-on support ensures that participants feel confident applying what they have learned [66]. To maintain momentum and provide additional support, the intervention research team will be available through email and phone, and booster sessions will be offered to address any emerging issues or to reinforce key concepts. These follow-up sessions will help solidify the training content and provide clarity on any challenges participants face [70].

Finally, technical assistance will be provided to help clinics build their capacity to implement the intervention successfully. This includes personalised facilitation for clinics that require additional help, ensuring that every clinic is fully supported throughout the implementation phase [67].

**Sustainable Implementation.** Sustainability is planned on two levels. First, after the sessions, participants will have access to and can consult the materials on the intervention’s website. Second, the intervention intends to encourage MHCPs to create online support groups within their communities, where they share their experiences on topics related to working with migrant clients and interpreters in linguistically challenging encounters.

#### Step 6: Planning evaluation.

The evaluation of the intervention will utilise a pre- and post-test quantitative design, which is a robust method for measuring intervention outcomes. Quantitative approaches are particularly effective for assessing the effectiveness of interventions, as they provide objective, measurable data [71]. The evaluation will focus on tests and analyses that align with the intervention’s specific goals and performance objectives, particularly emphasising individual-level behavioural changes. Success will be defined as significant improvements in performance objectives when compared to the baseline measurements [72].

#### Outcome evaluation.

The outcome evaluation will assess the extent to which the training leads to desired changes in MHCPs’ knowledge, confidence, perceived social norms, and behavioural intentions related to communication in linguistically diverse mental healthcare encounters.

The first part of the evaluation focuses on knowledge. This component is adapted from the mental health literacy scale (MHLS) [73] and the cultural competence self-assessment checklist (CCSA) [74]. It measures MHCPs’ understanding of interpreter roles, communication strategies, and the impact of language barriers on mental healthcare. Each item corresponds to specific learning outcomes covered during the training sessions.

The second section addresses self-efficacy and draws on Bandura’s self-efficacy scale [75]. It evaluates participants’ confidence in handling communication challenges in clinical practice, including their ability to work effectively with interpreters, use technological tools, and apply appropriate language when engaging with clients who face language barriers.

The third section focuses on perceived social norms and organisational expectations. Adapted from the theory of planned behaviour (TPB) [38], this part captures participants’ beliefs about the extent to which colleagues and organisational leadership support the use of interpreters and endorse culturally responsive communication practices.

The fourth component assesses behavioural intentions using TPB-based items. It examines MHCPs’ intentions to apply newly acquired skills in clinical practice, for instance, by using professional interpreters, adapting communication styles, and advocating for improved access to interpretation services and tools.

Finally, the evaluation includes several control variables to account for individual and contextual differences that may influence the intervention’s effectiveness. These include prior experience with interpreters, frequency of interpreter access in the workplace, general openness to learning, and familiarity with intercultural communication. These items are adapted from the language services utilisation scale (LSUS) [76], the intrinsic motivation inventory [51], and the cultural competence assessment (CCA) scale [77]. The complete questionnaire is provided in S2 Appendix.

#### Process evaluation.

The process evaluation focuses on the usability, satisfaction, and perceived relevance of the training, using items adapted from the questionnaire for professional training evaluation [78] and the reaction and learning levels of Kirkpatrick’s Model [79]. MHCPs will rate aspects such as the clarity of learning objectives, applicability of content, effectiveness of training methods (e.g., role-plays, discussions), and usefulness of supporting materials. This data will inform further refinement of the training intervention.

Measurements will be taken at three key time points: baseline (immediately before the training), immediately after the training (first post-implementation test), and three months after the intervention (secondary post-implementation test). This multi-phase evaluation design will enable a thorough assessment of both immediate and sustained impacts of the intervention, along with an analysis of its implementation effectiveness [29,80].

#### Data management plan.

All data will be managed in accordance with Utrecht University’s Research Data Management policy and GDPR requirements.

**Data collection:** Data will include de-identified feedback from mental healthcare providers through questionnaires and interviews.

**Data storage and security:** Data will be securely stored on Utrecht University’s protected servers, accessible only to the research team.

**Anonymisation:** All personal identifiers will be removed prior to analysis. Only coded, de-identified data will be retained.

**Data sharing:** Supporting materials (lesson plan and evaluation tools) are provided with this submission.

**Preservation:** Data will be preserved for a minimum of 10 years in Utrecht University’s institutional repository.

#### Ethical considerations.

The pilot testing of the intervention was conducted in compliance with ethical standards for research involving human participants. Ethical approval was obtained from Utrecht University’s Ethics Assessment Committee of the Faculty of Humanities (FEtC-H), under approval number 24-146-03. All participants were informed about the study’s purpose, procedures, and their rights, including voluntary participation and the ability to withdraw at any time without consequences. All data were handled confidentially in accordance with the General Data Protection Regulation (GDPR).

#### The status and timeline of the study.

The development of the training materials and study instruments has been completed. The pilot testing phase, including revisions to the training materials, has also been finalised.

Recruitment of participants will begin in October 2025. The implementation phase is scheduled to start in November 2025 and continue through February 2026. Data collection is expected to be completed by March 2026, with analysis and dissemination of findings anticipated by September 2026.

## Conclusion

This protocol outlines the development, implementation, and evaluation of a communication-focused training intervention designed to support mental health care providers in effectively collaborating with interpreters when working with migrant clients. By addressing common barriers related to language proficiency, cultural understanding, and undefined or vague professional roles [27,81], the intervention aims to strengthen provider competence and promote more equitable, patient-centred care. The study applies a theory-informed framework [28], employs validated measurement tools [73,75], and integrates a structured process evaluation [82] to ensure both rigour and relevance. Findings from this intervention will add to the growing body of evidence on culturally responsive mental health care and offer practical insights for future implementation in diverse clinical settings [29,83].

## Supporting information

S1 Appendix Training lesson plan. (DOCX)

S2 Appendix Evaluation. (DOCX)
