# Correction: When migrants do not speak the host country’s language but need mental health care: A protocol for developing a communication intervention

**DOI:** 10.1371/journal.pone.0357298

**Published:** 2026-08-28

**Authors:** Houda Al Kalaf, Christopher Jenks, Mike Mösko, Ted Sanders, Barbara Schouten

There is an error in references 57, 58, 60 and 61. The correct references are:

57. Ghahari S, et al. Development and pilot testing of a health education program to improve immigrants’ access to Canadian health services. BMC Health Serv Res. 2020 Apr 17;20(1):321. https://doi.org/10.1186/s12913-020-05180-y
58. Rhoda DA, et al. A Practical Guide to pilot Testing Community-Based Vaccination Coverage Surveys. Vaccines (Basel). 2023 Nov 28;11(12):1773. https://doi.org/10.3390/vaccines11121773
60. Shearer R, Davidhizar R. Using role play to develop cultural competence. Journal of Nursing Education J Nurs Educ. 2003 Jun 1;42(6):273–6. Available from: https://doi.org/10.3928/0148-4834-20030601-10
61. Wright SR, Homer M. Addressing the theory-practice gap in assessment. Perspectives on Medical Education. Perspect Med Educ. 2017 Jan 3;6(1):7–9. Available from: https://doi.org/10.1007/s40037-016-0323-z
