# Supplementary material for: When migrants do not speak the host country’s language but need mental health care: A protocol for developing a communication intervention
Source: PLoS One. 2025 Dec 5;20(12):e0338040. doi: 10.1371/journal.pone.0338040 (PMC12680212; doi:10.1371/journal.pone.0338040)
Supplement: S1 Appendix — (DOCX) [file pone.0338040.s001.docx]

**Supporting documents**

**S1 Appendix. Training lesson plan**

*Education programme- MHCPs*

Learning Outcomes

| At the end of Session 1, the MHCPs/participants will have: |
| --- |
| 1. Improved their awareness of the term "language barrier" in mental healthcare and the role of interpreters |
| 2. Improved their knowledge on how to work effectively with professional interpreters |
| 3. Improved their knowledge on how to work effectively with ad-hoc interpreters |
| 4. Improved their knowledge on how to work effectively with informal interpreters such as family and friends |
| 5. Improved their self-efficacy with regard to how to work with different types of interpreters and utilize alternative  communication strategies to bridge language barriers during linguistically challenged mental healthcare consultations (During the session, participants are only introduced to and familiarized with the role-playing method) |
| 6. Developed positive social norms, enhancing their ability to give and receive peer support when applying new  communication practices learned during the training. This includes supporting one another in performing new behaviours and integrating the knowledge acquired (During this session, participants are introduced to and familiarized with the  concept of mobilizing peer support) |

LESSON PLAN, Session 1, 3 hours

| Time | Trainer activity | Content | Participants activity | Material | Learning outcome |
| --- | --- | --- | --- | --- | --- |
| 35 minutes | **1. Introduction (5 min)** – Outline objectives, importance of language barriers, and participation expectations.  **2. Scenario Presentation (5 min)** – Assign groups, explain context, and highlight cultural nuances.  **3. Discussion (15 min)** – Note key insights, facilitate reflection, and connect research to practice.  **4. Presentations & Feedback (10 min)** – Groups share findings; trainer gives feedback and encourages dialogue.  **5. Conclusion**– Summarize takeaways, reinforce cultural awareness, and encourage practical application. | - The Term ‘Language Barrier’ – and What It Encompasses in Mental Healthcare Settings - Understanding the Role of Interpreters in Mental Healthcare: Benefits, Challenges, and the Need for Distinction - Interpreting in Mental Healthcare Settings | 1- I**ntroduction**– Listen, understand objectives, and reflect on language barriers.  2- **Group Work**– Analyze assigned scenarios, identifying key issues.  3- **Discussion**– Explore insights, answer reflection questions, and brainstorm solutions.  4- **Presentations**– Summarize findings, present strategies, and receive feedback.  5- **Conclusion**– Review key takeaways and consider real-world applications. | Interactive case study number 1, day 1.  The case study scenario and related discussion prompts | 1 |
| 30 minutes | 1- **Introduction (5 min)** – Discuss interpreter collaboration, set objectives, and reflect on past experiences through a “gallery walk.” Encourage participants to share insights.  2- **Case Scenario (10 min)** – Present a case study on interpreter collaboration, assign group discussions, and analyze pre-, during-, and post-session dynamics.  3- **Discussion & Solutions (10 min)** – Groups present findings; trainer highlights key takeaways and best practices for effective interpreter use.  4- **Conclusion (5 min)** – Summarize key lessons, present actionable strategies, and prompt participants to commit to one improvement for future sessions. | Knowledge for Collaborating with Different Types of Interpreters (professionals) | 1- **Introduction**– Listen to session objectives, reflect on interpreter experiences, share insights via post-it notes, and discuss key challenges.  2- **Case Analysis**– In groups, examine a case study, addressing pre-session prep, patient-centered communication, and post-session debriefing.  3- **Discussion & Solutions**– Develop and present solutions for assigned phases, engage in peer discussion, and receive feedback.  4- **Reflection**– Review key takeaways, identify one strategy to implement, and share insights. | Interactive case study number 2, day 1.  The case study scenario and related discussion prompts | 2 |
| 30 minutes | 1- I**ntroduction (5 min)** – Discuss challenges of ad-hoc interpreters, ethical risks, and engage participants with a reflection prompt.  2- **Case Scenario (10 min)** – Present a case involving an ad-hoc interpreter, assign group discussions on risks, improvements, and alternatives.  3- **Group Discussion (10 min)** – Groups share insights; trainer provides feedback on ethical concerns and best practices.  4- **Empathy Exercise (5 min)** – Reflect on patient perspectives when clinic staff act as interpreters, discussing emotional and ethical implications.  5- **Conclusion (5 min)** – Summarize key takeaways, present solutions, and prompt participants to commit to an improvement in their practice. | Knowledge for Collaborating with Different Types of Interpreters (ad-hoc) | **1- Introduction**– Understand objectives, reflect on ad-hoc interpreter experiences, and discuss challenges.  **2- Case Analysis**– In groups, examine a case study, identify risks, and discuss strategies for improvement.  **3- Group Discussion**– Present findings, compare strategies, and engage with trainer feedback.  **4- Empathy Exercise**– Reflect on patient perspectives and discuss ethical implications.  **5- Conclusion**– Summarize key takeaways, reflect on applications, and commit to an improvement. | Interactive case study number 3, day 1.  The case study scenario and related discussion prompts | 3 |
| 10 minutes | Break | | | | |
| 30 minutes | **1. Introduction (5 min)**  - Outline session focus and key challenges (confidentiality, power, bias, accuracy).  - Prompt participant reflections.  **2. Scenario Presentation (5 min)**  - Introduce Sara and Omar’s case, highlighting ethical concerns.  - Explain key risks (power imbalance, omissions, confidentiality).  **3. Group Activity (15 min)**  **- Analysis (7 min):** Guide discussions on risks and ethical concerns.  **- Solutions (8 min):** Support strategy development for ethical management.  **4. Debrief & Conclusion (5 min)**  - Discuss key takeaways and best practices.  - Wrap up with a reflection question and share resources. | Knowledge for Collaborating with Different Types of Interpreters (friend and family) | **1. Introduction**  - Reflect on experiences with informal interpreters.  - Engage in an opening discussion on challenges (confidentiality, power dynamics, bias).  **2. Scenario Analysis**  - Review and discuss the case of Sara and Omar.  - Identify key risks and ethical concerns.  **3. Group Activity**  **- Step 1:** Analyze the scenario in small groups.  **- Step 2:** Develop and propose strategies to address interpretation challenges.  **4. Debrief & Conclusion**  - Share insights from group discussions.  - Reflect on key takeaways and identify one strategy to apply in practice. | Interactive case study number 4, day 1.  The case study scenario and related discussion prompts | 4 |

| 30 minutes | 1- **Introduction (5 minutes)** – Explain the role-play’s purpose, objectives, and the importance of communication in mental health care.  2- **Selection of Participants (5 minutes)** – Choose participants to play the characters, and ensure they understand their characters’ backgrounds and challenges.  – Create a non-judgmental space for participants to engage freely in the role-play.  4- **Scene 1: Interpreter Dominance (5 minutes)** – Begin the role-play with Maha dominating the conversation, preventing Reem from fully expressing herself.  5- **Discussion (Scene 1) (7 minutes)** – Pause to discuss how Maha’s dominance affects Reem’s communication and how Dr. Jan could adjust his approach to involve Reem more actively.  6- **Scene 2: Improved Communication (5 minutes)** – Transition to Scene 2, where Dr. Jan guides Maha to be a more neutral interpreter and facilitates direct communication with Reem.  7- **Learning Outcomes (5 minutes)** – Summarize key takeaways: client-centered communication, family interpreter challenges, and empathy in therapy. | Role-Playing:  Self-efficacy Development | **1. Introduction-** Listen to the trainer’s objectives and context. And Reflect on experiences with family interpreters.  **2. Role Assignment** - Volunteer or be assigned roles of Dr. Jan, Maha, and Reem.  3. **Scene 1: Interpreter Dominance**  - Act out the role-play  - Observe how it affects Reem’s ability to communicate.  4. **Discussion**  - Share thoughts on the impact of interpreter dominance. - Discuss ways Dr. Jan could improve communication.  5. **Scene 2: Improved Communication-** Act out the role-play  6. **Learning Outcomes**- Summarize key takeaways on communication and cultural sensitivity. | 1st Role-play: The role-play scenario and related discussion prompts | 5 |
| --- | --- | --- | --- | --- | --- |
| 10 minutes | **Present the slides:**   - Setting Clear Objectives   Follow the notes attached to the slides | Introduction to the concept of mobilizing peer support | **Follow the presentation:**   - Active Listening - Constructive Feedback   Reflection and Learning | Slides of session 1 | 6 |

Learning Outcomes

| At the end of Session 2, the MHCPs/participants will have: |
| --- |
| 1 Improved their knowledge on how to utilize alternative communication strategies to mitigate language barriers. |
| 2 Improved their self-efficacy with regard to how to work with different types of interpreters and utilize alternative communication  strategies to bridge language barriers during linguistically challenged mental healthcare consultations |
| 3 Developed positive social norms, enhancing their ability to give and receive peer support when applying new communication  practices learned during the training. This includes supporting one another in performing new behaviours and integrating the knowledge acquired |

LESSON PLAN, Session 2, 3 hours

| Time | Trainer activity | Content | Participants activity | Material | Learning  outcome |
| --- | --- | --- | --- | --- | --- |
| 10 minutes | **Present the slides:**   - Setting Clear Objectives - Follow the notes attached to the slides | Skills for assessing language proficiency of the client | **Follow the presentation:**   - Active Listening - Constructive Feedback - Reflection and Learning | Slides- session 2 | 1 |
| 30 minutes | **1. Introduction (5 min)**  - Introduce the case study and objectives.  - Prompt brief discussion on language barriers.  **2. Exploring Strategies (20 min)**  - Guide discussion on communication barriers.  - Introduce and discuss three approaches: Visual Tools (charts, images), Non-Verbal Cues (gestures, body language), and Technology (apps, digital tools)  - Facilitate brainstorming on combining strategies**.**  **3. Reflection & Wrap-Up (5 min)**  - Participants note one strategy to apply.  - Summarize key insights and share resources. | Skills for using alternative communication strategy approaches | **1. Introduction-** Reflect on challenges in communication without an interpreter.  **2. Exploring Strategies-**  - Discuss language barriers and their impact on treatment.  - Brainstorm and share alternative communication strategies.  - Engage in discussion on visual tools, non-verbal cues, and technology.  - Consider how to combine strategies for effective communication.  **3. Reflection & Wrap-Up-**  - Write down one strategy they feel confident using.  - Participate in a brief discussion on key takeaways. | Interactive case study number 1, day 2.  The case study scenario and related discussion prompts | 1 |

| 45 minutes | **Present the slides:**   - Setting Clear Objectives   Follow the notes attached to the slides | A Deeper Look at Alternative Communication  Strategies:  Smart Communication  Tools in Mental Healthcare | **Follow the presentation:**   - Active Listening - Constructive Feedback   Reflection and Learning | Slides- session 2 | 1 |  |
| --- | --- | --- | --- | --- | --- | --- |
| 45 minutes | 1. **Introduction (5 minutes)** Explain the role-play’s purpose, objectives  2. **Selection of Participants (5 minutes)**- Choose participants to play the characters, and ensure they understand their characters’ backgrounds and challenges.  3. **Scene 1: Initial Interaction & Miscommunication (5 minutes)**  4. **Audience/participants Discussion (5 minutes)**- Facilitate a discussion on what went wrong  **5. Scene 2: Improved Communication (10 minutes)**-  6. **Debrief and Discussion (10 minutes)** | Role-Playing:  Self-efficacy Development | 1. **Introduction & Briefing-** Listen to the trainer’s introduction and role-play scenario.  2. **Scene 1: Initial Interaction-**Observe the interaction, noting communication breakdowns and challenges and recognize key issues in working with interpreters  3. **Audience Discussion**- Identify barriers to effective communication and their impact on patient care.  4. **Scene 2: Improved Communication-** See how strategies improve communication and lead to better outcomes.  5. **Debrief and Discussion-** Discuss the changes in approach and their impact on the session and patient care. | 2^nd^ Role-play: The role-play scenario and related discussion prompts | 2 | |
| 15 minutes | Break | | | | | |
| 45 minutes | 1. **Introduction (5 minutes)** – Explain the role-play’s purpose, objectives  2. **Selection of Participants (5 minutes)**- Choose participants to play the characters, and ensure they understand their characters’ backgrounds and challenges.  2. **Facilitate Scene 1(10 minutes):** Miscommunication and failure to make a diagnosis  4. **Facilitate Scene 2(10 minutes:** Culturally attuned communication  5. **Post-Session Debrief (10 minutes)**- Reinforce the importance of cultural sensitivity and clear communication, and provide guidance on how participants can implement these practices in real-life scenarios.  6. **Closing Remarks and Reflection (5 minutes)**- Trainer summarizes key takeaways from the role-play. | Role-Playing:  Self-efficacy Development | 1. **Introduction & Briefing-** Listen to the trainer’s introduction and role-play scenario.  2. **Scene 1: Initial Interaction-**Observe the interaction, noting communication breakdowns and challenges and recognize key issues in working with inexperienced interpreter.  3. **Audience Discussion**- Identify barriers to effective communication and their impact on patient care.  4. **Scene 2: Improved Communication-** See how strategies improve communication and lead to better outcomes.  5. **Debrief and Discussion-** Discuss the changes in approaches and their impact on the session and patient care. | 3^rd^ Role-play: The role-play scenario and related discussion prompts | 2 | |

| 5 minutes | **Present the video:**   - Setting Context - Guiding Focus - Facilitating Active Engagement - Connecting to Learning Objective - Encouraging Reflection and   Action | Social norms development: testimony from a role model | **Watch the video:**   - Active Viewing - Reflective Thinking - Application of Insights | A self-recorded video of a local MHCP promoting the acquisition and application of effective skills for working with interpreters. | 3 |
| --- | --- | --- | --- | --- | --- |
| 30 minutes | **1. Introduction (5 min)** – Set expectations for peer support and facilitate a brief discussion on past experiences.  **2. Case Study (10 min)** – Present Dr. Miller’s hesitation and Dr. Patel’s challenge in offering support.  **3. Group Discussion (10 min)** – Guide small groups to explore solutions for creating norms, giving support, and normalizing help-seeking.  **4. Debrief (5 min)** – Highlight key takeaways and reinforce the value of peer collaboration.  **5. Action Plan (5 min)** – Help participants define concrete steps to embed peer support in their practice. | Social norms development: Skills for giving /receiving peers’ support. | **1. Reflection (5 min)** – Share experiences of receiving or giving peer support in their professional development.  **2. Case Study Analysis (10 min)** – Discuss Dr. Miller’s and Dr. Patel’s challenges and identify potential solutions.  **3. Group Problem-Solving (10 min)** – Brainstorm strategies for establishing peer support, giving constructive feedback, and encouraging help-seeking.  **4. Debrief (5 min)** – Share insights and reflect on how peer support can strengthen team collaboration.  **5. Action Planning (5 min)** – Develop practical steps to integrate peer support into their work environment. | Interactive case study number 2, day 2.  The case study scenario and related discussion prompts | 3 |
