# Supplementary material for: When migrants do not speak the host country’s language but need mental health care: A protocol for developing a communication intervention
Source: PLoS One. 2025 Dec 5;20(12):e0338040. doi: 10.1371/journal.pone.0338040 (PMC12680212; doi:10.1371/journal.pone.0338040)
Supplement: S2 Appendix — (DOCX) [file pone.0338040.s002.docx]

**S2 Appendix. Evaluation**

Intervention Evaluation Questionnaire

**Part 1: Section 1**

Instructions: Please indicate the extent to which you agree or disagree with the following statements.

Scale:

1: Strongly Disagree

2: Disagree

3: Neither agree nor disagree

4: Agree

5: Strongly Agree

| Statement | 1 | 2 | 3 | 4 | 5 |
| --- | --- | --- | --- | --- | --- |
| I am aware that the presence of an interpreter affects the therapeutic process and that it is important to manage this dynamic |  |  |  |  |  |
| I understand that the interpreter should have clear expectations regarding their roles and responsibilities before a session begins. |  |  |  |  |  |
| I am aware that interpreters should not offer their opinions on the client's situation but only relay the client’s words faithfully. |  |  |  |  |  |
| I am aware that language barriers can obscure the nuances of a client’s emotional expressions, potentially hindering an accurate understanding of their mental health concerns. |  |  |  |  |  |

| Statement | 1 | 2 | 3 | 4 | 5 |
| --- | --- | --- | --- | --- | --- |
| I understand that professional interpreters may struggle to capture emotional tone or non-verbal cues (especially in over-the-phone interpretation), which can be important in a mental health setting. |  |  |  |  |  |
| I know that using professional interpreters in mental health sessions may create a sense of discomfort for some clients, as they may feel their personal information is being shared with a third party. |  |  |  |  |  |
| I understand that professional interpreters are more suitable than family members when it comes to interpreting at mental health sessions. |  |  |  |  |  |

| Statement | 1 | 2 | 3 | 4 | 5 |
| --- | --- | --- | --- | --- | --- |
| **I am aware of the potential risks of using non-professional interpreters in mental health sessions, such as misinterpretation or emotional involvement** |  |  |  |  |  |
| **I recognise that non-professional interpreters, such as family members, may face challenges in maintaining professional boundaries in therapy sessions.** |  |  |  |  |  |
| **I understand that using a non-professional interpreter could raise concerns about confidentiality or impartiality in certain situations.** |  |  |  |  |  |

| Statement | 1 | 2 | 3 | 4 | 5 |
| --- | --- | --- | --- | --- | --- |
| **I recognise that using technology for interpretation in mental health sessions can ensure faster access to services, particularly for clients in rural or underserved areas.** |  |  |  |  |  |
| **I am aware that the use of technology-based interpretation tools should be used with caution, especially in terms of privacy and security in mental health settings.** |  |  |  |  |  |
| **I understand that interpretation tools, e.g., Google Translate and telephone interpreting services, are commonly used but may not be as effective for complex or sensitive mental health counselling sessions.** |  |  |  |  |  |

**Part 1: Section 2**

Instructions: On a scale of 1 (Not at all confident) to 5 (Very confident), please rate your confidence in the following areas:

Scale:

1: Not at all confident

2: Slightly confident

3: Moderately confident

4: Very confident

5: Extremely confident

| Statement | 1 | 2 | 3 | 4 | 5 |
| --- | --- | --- | --- | --- | --- |
| Communicating effectively with a family member or a friend as an interpreter. |  |  |  |  |  |
| Communicating clearly and professionally when using a **professional interpreter** |  |  |  |  |  |
| Using **online materials** or **visual aids** effectively to supplement communication with clients with whom I have a language barrier |  |  |  |  |  |
| Adapting to **technical difficulties** or **communication breakdowns** when working with a **professional interpreter over the phone** and finding ways to maintain the flow of the session. |  |  |  |  |  |
| Using the correct medical terminology in a way that a family member, friend, clinic worker, or professional interpreter can understand and explain to the client. |  |  |  |  |  |

**Part 1: Section 3**

Instructions: Please indicate the extent to which you agree or disagree with the following statements.

Scale:

1: Strongly Disagree

2: Disagree

3: Neither agree nor disagree

4: Agree

5: Strongly Agree

| Statement | 1 | 2 | 3 | 4 | 5 |
| --- | --- | --- | --- | --- | --- |
| I feel that my colleagues generally support the use of professional interpreters when working with clients who do not speak my language. |  |  |  |  |  |
| The board of my organisation actively promotes the integration of best practices for interpreter-mediated therapy into our professional standards. |  |  |  |  |  |
| I believe that using professional interpreters improves the quality of care and outcomes for clients with language barriers. |  |  |  |  |  |
| I feel comfortable advocating for the use of professional interpreters in clinical settings. |  |  |  |  |  |
| I believe that using interpreters effectively is a crucial skill for mental health providers. |  |  |  |  |  |

**Part 1: Section 4**

24- I intend to use professional interpreters whenever possible in my clinical practice.

Extremely Unlikely

Unlikely

Neutral

Likely

Extremely Likely

25- I intend to establish clear communication guidelines with interpreters before each session, including the importance of confidentiality and neutrality

Definitely False

Probably False

Neutral

Probably True

Definitely True

26- I intend to use technology-assisted interpretation tools (e.g., video remote interpreting and translation apps) when professional interpreters are unavailable.

Extremely Unlikely

Unlikely

Neutral

Likely

Extremely Likely

27- I intend to adjust my speech and use plain language to ensure clarity when working with non-professional interpreters.

Definitely False

Probably False

Neutral

Probably True

Definitely True

28- I plan to advocate for improved access to interpreters and alternative communication resources in my workplace.

Strongly Disagree

Disagree

Neutral

Agree

Strongly Agree

**Part 2:**

29- How often have you worked with interpreters in mental health settings before attending this training?

Always

Frequently

Occasionally

Rarely

Never

30- How often do you have access to professional interpreters in your current workplace?

Always

Frequently

Occasionally

Rarely

Never

31- How would you describe your general attitude toward learning new skills and practices in your profession?

Very open

Open

Neutral

Reluctant

Very reluctant

32- How familiar are you with the cultural differences that can influence communication in therapy sessions with interpreters?

Very familiar

Somewhat familiar

Neutral

Somewhat unfamiliar

Not familiar at all

**Part 3:**

Instructions: Please indicate the extent to which you agree or disagree with the following statements.

Scale:

1: Strongly Disagree

2: Disagree

3: Neither agree nor disagree

4: Agree

5: Strongly Agree

| Statement | 1 | 2 | 3 | 4 | 5 |
| --- | --- | --- | --- | --- | --- |
| This training provided me with practical, applicable knowledge that will enhance my ability to work effectively with interpreters in clinical settings. |  |  |  |  |  |
| The training format, which included lectures, role-playing, and discussions, was effective and helped me better understand how to apply these skills in practice. |  |  |  |  |  |
| After the training, I feel more confident in managing the complexities involved in working with interpreters, including ethical concerns and cultural considerations. |  |  |  |  |  |
| The trainer communicated the training objectives clearly. |  |  |  |  |  |
| The materials and resources provided during the training were clear and easy to understand. |  |  |  |  |  |
